# Preserved Daily Function Despite Significant Muscle Atrophy and Strength Deficits: A Matched‐Cohort Study of Iliopsoas Release for Ischiofemoral Impingement

**DOI:** 10.1111/os.70295

**Published:** 2026-04-22

**Authors:** Yu‐Peng Duan, Hao Sun, Hong‐Jie Huang, Yan Xu, Xiao‐Dong Ju, Jian‐Quan Wang

**Affiliations:** ^1^ Department of Sports Medicine Peking University Third Hospital, Institute of Sports Medicine of Peking University Beijing China

**Keywords:** Goutallier classification, iliopsoas release, ischiofemoral impingement, isokinetic strength, muscle atrophy

## Abstract

**Objective:**

The arthroscopic management of concomitant ischiofemoral impingement (IFI) and femoroacetabular impingement (FAI) frequently necessitates iliopsoas release; however, concerns regarding potential iatrogenic muscle atrophy and strength deficits remain unresolved. Therefore, the purpose of this study was to evaluate the clinical outcomes, volumetric changes, muscle morphology, and objective isokinetic muscle strength in patients undergoing arthroscopic treatment for concomitant IFI and FAI involving iliopsoas release.

**Methods:**

This retrospective matched‐cohort study analyzed patients treated between January 2019 and January 2020. It included 15 female patients (IFI + FAI group) who underwent arthroscopic IFI decompression and iliopsoas release, and 15 propensity‐matched patients (Isolated FAI group) treated for FAI alone. At a minimum 2‐year follow‐up, patient‐reported outcomes (PROs) were assessed. Muscle morphology was evaluated on MRI using 3D volumetric reconstruction for muscle volume, and the Goutallier classification was used to assess fatty infiltration at three standardized anatomical levels. Functional recovery was objectively measured using isokinetic hip flexor and extensor strength testing at 60°/s and 180°/s, comparing the involved hip to the uninvolved contralateral side. Statistical analyses included paired *t*‐tests, Mann–Whitney U tests, and Wilcoxon signed‐rank tests.

**Results:**

Both groups demonstrated significant improvements in PROs postoperatively (*p* < 0.001). Volumetric analysis revealed a significant reduction in iliopsoas muscle volume (25.5% decrease) in the IFI + FAI group postoperatively. Despite this volumetric reduction, postoperative MRI demonstrated preservation of muscle quality, as evidenced by low Goutallier grades (Grade < 1) and no significant difference in fatty infiltration compared to the control group (*p* > 0.05). Functionally, isokinetic testing demonstrated significant deficits in peak flexion torque compared to the healthy contralateral side (*p* < 0.001). Notably, while outcomes for activities of daily living were satisfactory, a statistically significant difference was observed in the Substantial Clinical Benefit (SCB) achievement rate regarding sports function between the groups.

**Conclusion:**

Iliopsoas release resulted in significant strength deficits, but no significant changes in muscle morphology were observed. However, patient‐reported outcomes (PROs) showed improvement, with no significant increase in fatty infiltration, suggesting that functional recovery may occur despite structural deficits.

## Graphical abstract

This matched‐cohort study reveals that iliopsoas release causes significant atrophy and weakness without fatty degeneration. While daily function is well‐preserved, the lower rate of achieving substantial clinical benefit in sports suggests potential limitations for high‐demand athletes.

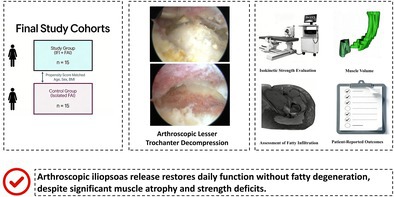

## 1 Introduction

Ischiofemoral impingement (IFI) is an extra‐articular hip pathology characterized by the pathological narrowing of the space between the lateral aspect of the ischial tuberosity and the medial aspect of the lesser trochanter [1, 2]. This narrowing creates a mechanical conflict often described as the “nutcracker phenomenon,” which entraps the quadratus femoris (QF) muscle and potentially the adjacent sciatic nerve, leading to edema, fatty infiltration, and debilitating deep gluteal pain [3, 4]. While historically associated with complications following total hip arthroplasty, contemporary literature has elucidated its significant prevalence in native hips. Notably, IFI frequently coexists with femoroacetabular impingement (FAI), likely due to shared morphological predispositions such as excessive femoral anteversion and valgus neck‐shaft angles [5, 6].

Surgical management of symptomatic IFI typically involves endoscopic decompression of the ischiofemoral space via lesser trochanteroplasty, which has demonstrated favorable clinical outcomes [7, 8]. In the setting of concomitant IFI and FAI, the surgical strategy is complex [9, 10]. To adequately visualize and resect the lesser trochanter, or to address concomitant internal snapping hip syndrome, an iliopsoas release (tenotomy) is frequently performed. This procedure serves a dual purpose: it resolves the snapping component and facilitates safe surgical access to the impingement zone posterior to the lesser trochanter [11].

However, the iliopsoas complex is the primary hip flexor and a critical dynamic stabilizer of the hip joint. Consequently, the routine inclusion of iliopsoas release remains a subject of debate among hip preservation surgeons [12, 13]. Concerns persist regarding potential iatrogenic sequelae, specifically muscle atrophy and permanent loss of hip flexion strength [14, 15]. While patient‐reported outcomes (PROs) following IFI surgery are favorable, few studies have objectively quantified postoperative iliopsoas integrity using MRI‐based volumetry or isokinetic strength testing [16].

Therefore, the purpose of this study was to evaluate the clinical outcomes, MRI‐based muscle morphology (fatty infiltration and volume), and objective isokinetic muscle strength in patients undergoing combined arthroscopic treatment for IFI and FAI involving iliopsoas release. We hypothesize that iliopsoas release will lead to structural and strength deficits, but that PROs will improve without significant fatty infiltration.

## 2 Methods

### 2.1 Patient Selection

This retrospective study was approved by the Institutional Review Board (IRB) (Approval No. M2020454) and analyzed prospectively collected data of patients undergoing hip arthroscopy by a single senior surgeon between January 2019 and January 2020. The study cohort consisted of patients diagnosed with symptomatic IFI concomitant with FAI [17]. It is important to note that during the study period, all patients undergoing surgical treatment for IFI at our institution were female. To reflect this clinical reality and eliminate sex‐based heterogeneity in muscle parameters, this study exclusively included female patients [18]. The diagnosis of IFI was established based on a standardized protocol: (1) deep gluteal pain and tenderness over the ischiofemoral space; (2) positive provocative physical examinations, specifically the IFI test and long‐stride walking test [9, 19]; and (3) MRI evidence of ischiofemoral space (IFS) (< 15 mm) or QF muscle edema/fatty infiltration [2, 20].

Inclusion criteria were: (1) age 18–55 years; (2) confirmed diagnosis of IFI and FAI; (3) failure of conservative treatment for > 3 months; and (4) minimum 2‐year follow‐up. Exclusion criteria included: (1) Tönnis grade > 1 osteoarthritis; (2) hip dysplasia (LCEA < 18°); (3) prior ipsilateral hip surgery; (4) incomplete radiographic or MRI data; or (5) subsequent revision surgery.

To isolate the effect of the surgical intervention, a control group of patients with isolated FAI (without IFI) was identified. Propensity score matching (PSM) was utilized to select matched controls based on age, sex, and Body Mass Index (BMI).

### 2.2 Patient‐Reported Outcomes (PROs)

PROs were collected preoperatively and at a minimum of 2 years postoperatively to evaluate clinical improvement. The specific outcome measures included the modified Harris Hip Score (mHHS), the Hip Outcome Score–Activities of Daily Living (HOS‐ADL) subscale and HOS–Sport‐Specific Subscale (HOS‐SS), the International Hip Outcome Tool (iHOT‐12), and the Visual Analog Scale (VAS) for pain. To assess the proportion of patients with clinically meaningful improvements, we utilized two validated patient‐reported outcome metrics: the minimal clinically important difference (MCID), the patient acceptable symptomatic state (PASS), and substantial clinical benefit (SCB). MCID thresholds were derived via a distribution‐based approach defined as one‐half the standard deviation (SD) of preoperative scores within the study cohort. For PASS, we employed an anchor‐based strategy patients responded to the dichotomous anchor question: “Considering all your daily activities, current pain levels, and functional limitations, do you consider your current condition satisfactory?” [21, 22]. Throughout the entire follow‐up period, any complications and subsequent reoperations were meticulously recorded.

### 2.3 Measurement of the IFS and QFS

The measurement method followed was as described by Maras Özdemir et al. [23]. IFS is the distance from ischial tuberosity to iliopsoas tendon or lesser trochanter (LT), and QFS is the gap between hamstring tendons and iliopsoas tendon or LT [24].

### 2.4 MRI Image Processing and Quantification of Muscle Volume

Muscle volumes were quantified using 3D Slicer software (version 4.11.0) based on preoperative and postoperative DICOM data. Magnetic resonance imaging examinations were performed using a 3.0‐Tesla scanner (GE Healthcare, Chicago, IL, USA). Muscle morphology and volume were assessed primarily on axial proton density (PD)‐weighted sequences to ensure optimal soft‐tissue contrast and differentiation between muscle fibers and intermuscular fat. The slice thickness was set at 3 mm. Following data import, regions of interest were isolated, and axial planes were segmented with manual alignment to the volumetric plane to ensure orthogonal measurements [25].

**FIGURE 1:**
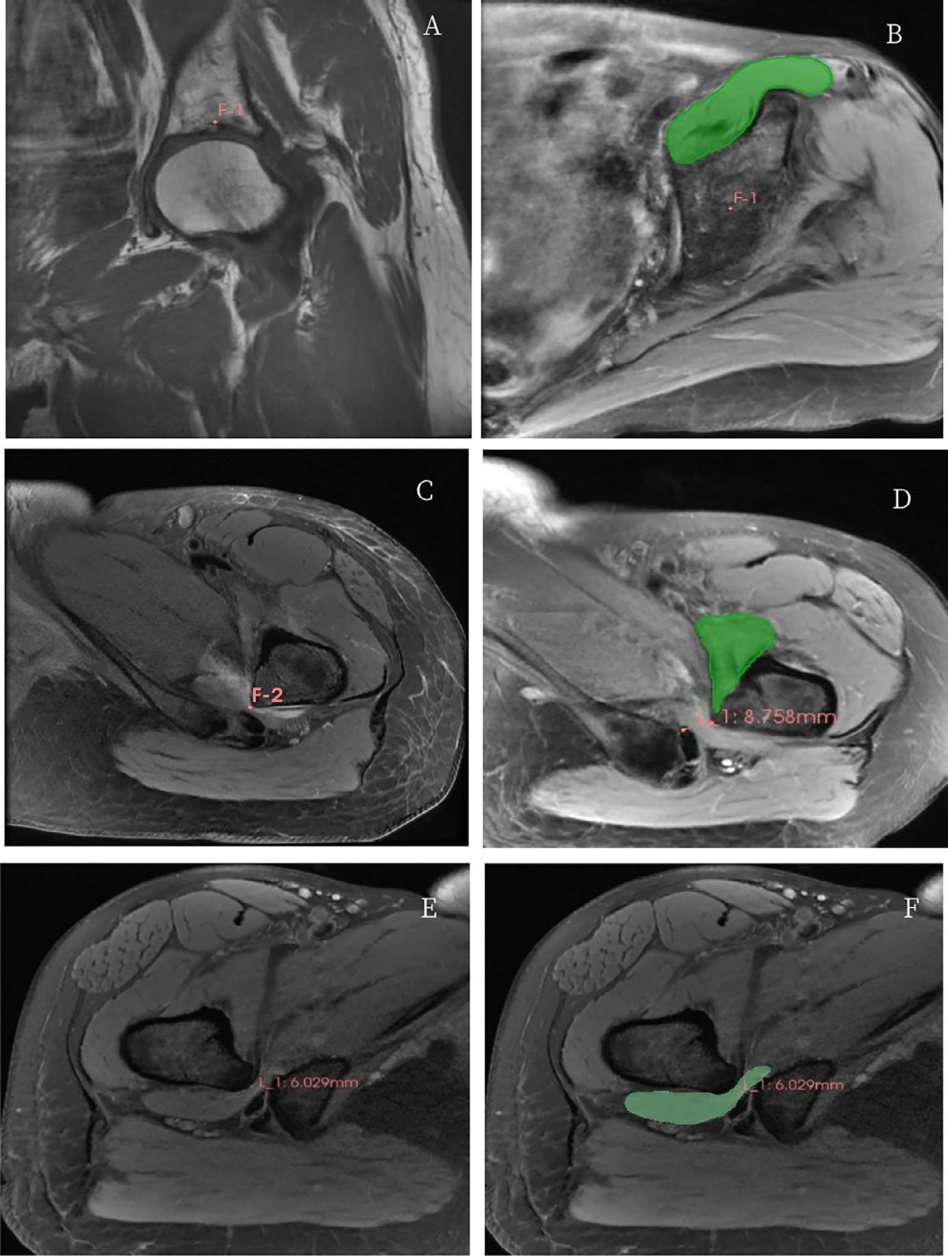
MRI for anatomical delineation of the iliopsoas and quadratus femoris muscles. (A) Proximal border of iliopsoas. (B) Proximal CSA. (C) Distal border. (D) Distal CSA. (E) QFS measurement. (F) QF muscle CSA. Abbreviations: CSA, cross‐sectional area; IFS, ischiofemoral space; QFS, quadratus femoris space; QF, quadratus femoris.

Iliopsoas Muscle Measurement: A standardized measurement protocol was established to define the proximal and distal boundaries of the iliopsoas muscle. The proximal boundary was defined at the level of the acetabular rim, corresponding to the maximum coronal diameter of the femoral head (Figure 1A,B). The distal boundary was set at the axial slice demonstrating the narrowest ischiofemoral space (IFS) (Figure 1D), consistent with previous protocols for IFI assessment [2]. Muscle boundaries were manually contoured using the segmentation tool (paint effect), with strict exclusion of cortical bone and distinct intramuscular fat (Figure 1C). To ensure efficiency and accuracy, manual segmentation was performed every two slices, followed by a semi‐automated interpolation algorithm and subsequent manual correction of the generated contours [26].

Quadratus Femoris (QF) Muscle Measurement: Given the anatomical characteristics of the QF, its volume was calculated from five consecutive axial slices. To ensure longitudinal comparability, the corresponding postoperative slices were identified by strictly matching the preoperative iliac and ischial bone morphology (Figure 1), a technique adapted from established muscle cross‐sectional area (CSA) measurement methodologies [27]. 3D reconstructions of the segmented muscles were generated for volumetric analysis (Supporting Information: Supplementary Material 1).

Reliability and Normalization: Two independent raters assessed the interrater reliability by measuring muscle volumes preoperatively and postoperatively. To account for variations in patient body habitus, raw muscle volumes were normalized to BMI. The Normalized Muscle Volume (NMV) was calculated as: Volume (mm^3^)/BMI (kg/m^2^) [28].

### 2.5 Isokinetic Muscle Strength Evaluation

At the final follow‐up, isokinetic muscle strength testing was performed in all patients in the IFI + FAI group at final follow‐up to assess the functional impact of the muscle's atrophy. Patient‐reported outcomes were also collected. Bilateral hip flexor and extensor strength was evaluated using a Biodex System 4 dynamometer (Biodex Medical Systems, Shirley, NY, USA) via a concentric contraction protocol.

Subjects were positioned supine on the dynamometer and secured to limit compensatory movements. Pelvic stability was achieved using a loop strap, with contralateral limbs restrained by padded restraints. The dynamometer axis aligned with the greater trochanter of the femur, and the resistance pad was positioned near the knee on the thigh.

Prior to testing, subjects completed a standardized 5‐min low‐intensity warm‐up followed by dynamic stretching of hip and thigh muscles. Hip range of motion was limited to the patient's pain‐free active movement, with limb and equipment weight compensated using gravity correction.

To minimize learning effects, the unaffected hip was tested first. Two angular velocities were employed: 60°/s (five maximum repetitions) to assess peak force, and 180°/s (10 maximum repetitions) to evaluate muscle power and endurance. A 60‐s rest interval was maintained between sets. Data were analyzed using Biodex Advantage BX software, with reported outcomes including peak torque (N·m), weight‐normalized peak torque (%), total work (J), and average power (W).

### 2.6 MRI Assessment of Fatty Infiltration

Fatty infiltration of the iliopsoas muscle was evaluated on axial T1‐weighted MRI sequences using the Goutallier classification system (Grade 0–4) [16].

**FIGURE 2:**
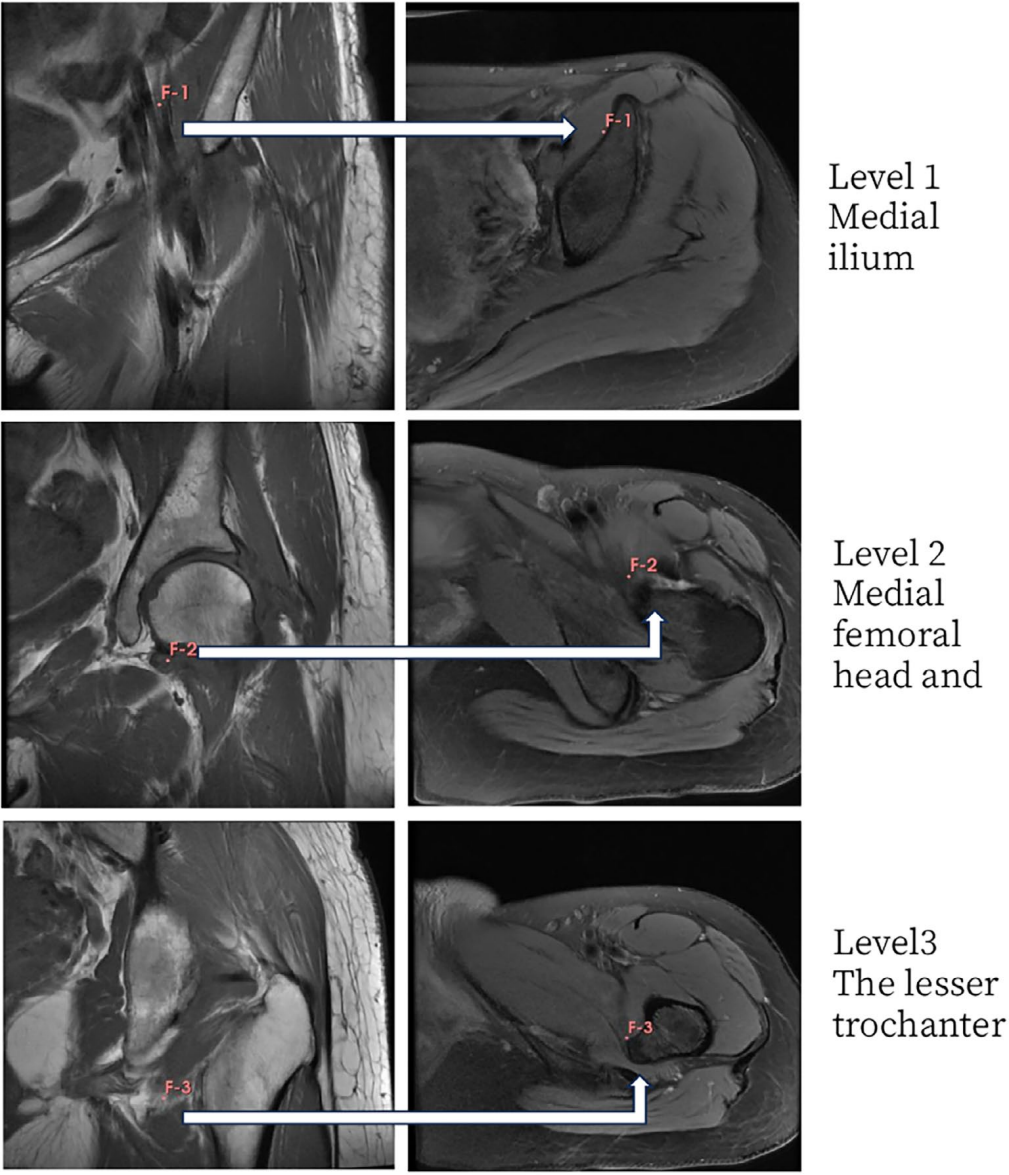
Standardized axial T1‐weighted MRI levels for iliopsoas assessment. Level 1: Medial ilium. Level 2: Medial femoral head and neck. Level 3: Lesser trochanter.

The assessment protocol was adapted from the methodology described by Kaniewska et al. [16]. Since the proximal lumbar levels (L4/L5) described in the original protocol were typically outside the field of view (FOV) of standard hip MRI, we established a standardized three‐level protocol based on visible anatomical landmarks to ensure consistent evaluation (Figure 2):

1. Level 1 (Proximal): Medial Ilium Level. The axial slice selected at the level of the medial aspect of the ilium. This level represents the proximal muscle belly of the iliopsoas complex within the iliac fossa.
2. Level 2 (Middle): Medial Femoral Head and Neck Level. The axial slice at the level of the medial femoral head and neck. This level captures the musculotendinous unit as it traverses the hip joint capsule.
3. Level 3 (Distal): Lesser Trochanter Level. The axial slice at the level of the lesser trochanter, representing the distal insertion site and the primary zone of impingement.

Two fellowship‐trained musculoskeletal radiologists independently graded the fatty infiltration at these three levels.

### 2.7 Surgical Methods

Surgical procedures were performed supine on a hip traction table. After 8–10 mm distraction and intra‐articular assessment with 15–20 min of traction, the capsule was sutured, traction released, and perineal post removed. All IFI surgical steps were completed traction‐free.

**FIGURE 3:**
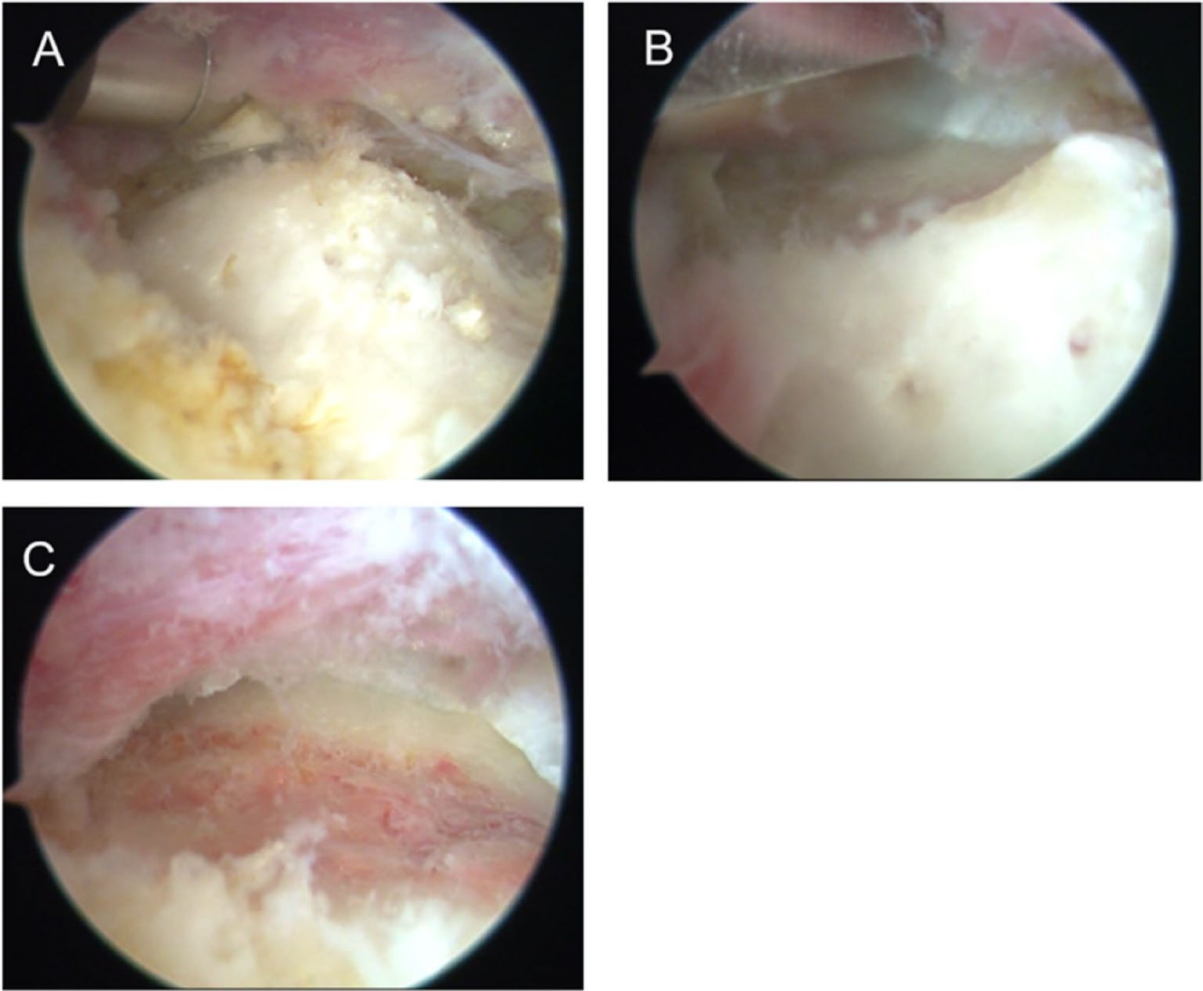
Endoscopic views of iliopsoas release and lesser trochanter resection. (A) Radiofrequency transection of iliopsoas tendon. (B) Lesser trochanter resection with arthroscopic burr. (C) Postresection view.

The hip was externally rotated and flexed to 30° to relax the iliopsoas tendon, allowing the lesser trochanter to shift from a posterior to an anterior position [29]. This follows the original technique described by Ilizaliturri and Camacho‐Galindo [14] for iliopsoas tendon resection in cases of snapping hips [30]. The procedure involved creating two anterolateral portals, with the working portal 4 cm below the optical portal. A cannulated needle was advanced through the inferior portal under fluoroscopic guidance to the lesser trochanter, followed by an arthroscopic cannula. The iliopsoas tendon, visible as a bright white structure, was released using a hooked radiofrequency probe (Figure 3A). An arthroscopic burr was then used to resect the lesser trochanter under fluoroscopic control (Figure 3B,C), estimating the distance to the ischium. Intraoperative dynamic tests confirmed IFI decompression. All procedures were performed by a senior surgeon.

### 2.8 Postoperative Rehabilitation

Effective postoperative rehabilitation is crucial for the successful management of IFI and FAI. Gradual progression in rehabilitation is essential to avoid inflammatory flares [31]. All patients followed a standardized rehabilitation protocol with physiotherapist guidance. Ankle pump exercises began 1‐day postsurgery, with 1–2 days of nonweight‐bearing. Weight‐bearing and painless passive range‐of‐motion exercises started within 2–7 days. From 10 days to 3 weeks, patients performed passive and active hip movements, including isometric exercises. Single‐legged balance training began at 3 weeks, and active hip movements were introduced at 4 weeks. Full weight‐bearing was allowed after 6 weeks, with gradual resumption of normal activities. High‐level activities were reintroduced between 3 and 6 months postoperatively [32].

### 2.9 Statistical Analysis

Intraclass correlation coefficients (ICCs) were calculated using a 2‐way random model with absolute agreement to evaluate interrater reliability and 95% confidence intervals (CIs) for muscle volume measurements. Statistical analyses were conducted using SPSS software (version 26.0, SPSS, Chicago, IL). Statistical analysis was performed using the Shapiro–Wilk test to assess the normality of the data distribution. For normally distributed variables, paired *t*‐tests were used to compare pre‐ and postoperative measurements. For nonnormally distributed variables, the Mann–Whitney U test was employed for between‐group comparisons, and the Wilcoxon signed‐rank test was used for within‐group comparisons. A *p*‐value of less than 0.05 was considered statistically significant. Additionally, to assess the effect size, Cohen's *d* was calculated for paired comparisons.

## 3 Results

To identify eligible patients, we reviewed data from all patients who underwent hip arthroscopy at our institution between January 2019 and January 2020. Among 295 patients meeting inclusion criteria, 25 were excluded due to predefined exclusion criteria: Tönnis grade > 1, acetabular dysplasia, prior hip surgery, insufficient follow‐up, or missing MRI and clinical data. The remaining 270 patients constituted the eligible cohort, all of whom were diagnosed with FAI. Among them, 15 female patients were additionally diagnosed with IFI and met all inclusion criteria (IFI + FAI group).

To establish a comparable control group, we identified 135 female patients who underwent hip arthroscopy for isolated acetabular impingement during the same period. PSM using a 1:1 nearest neighbor algorithm was performed to minimize selection bias, with matching variables including age, sex (all female), and BMI. This process yielded a matched isolated hip impingement group (*n* = 15).

**FIGURE 4:**
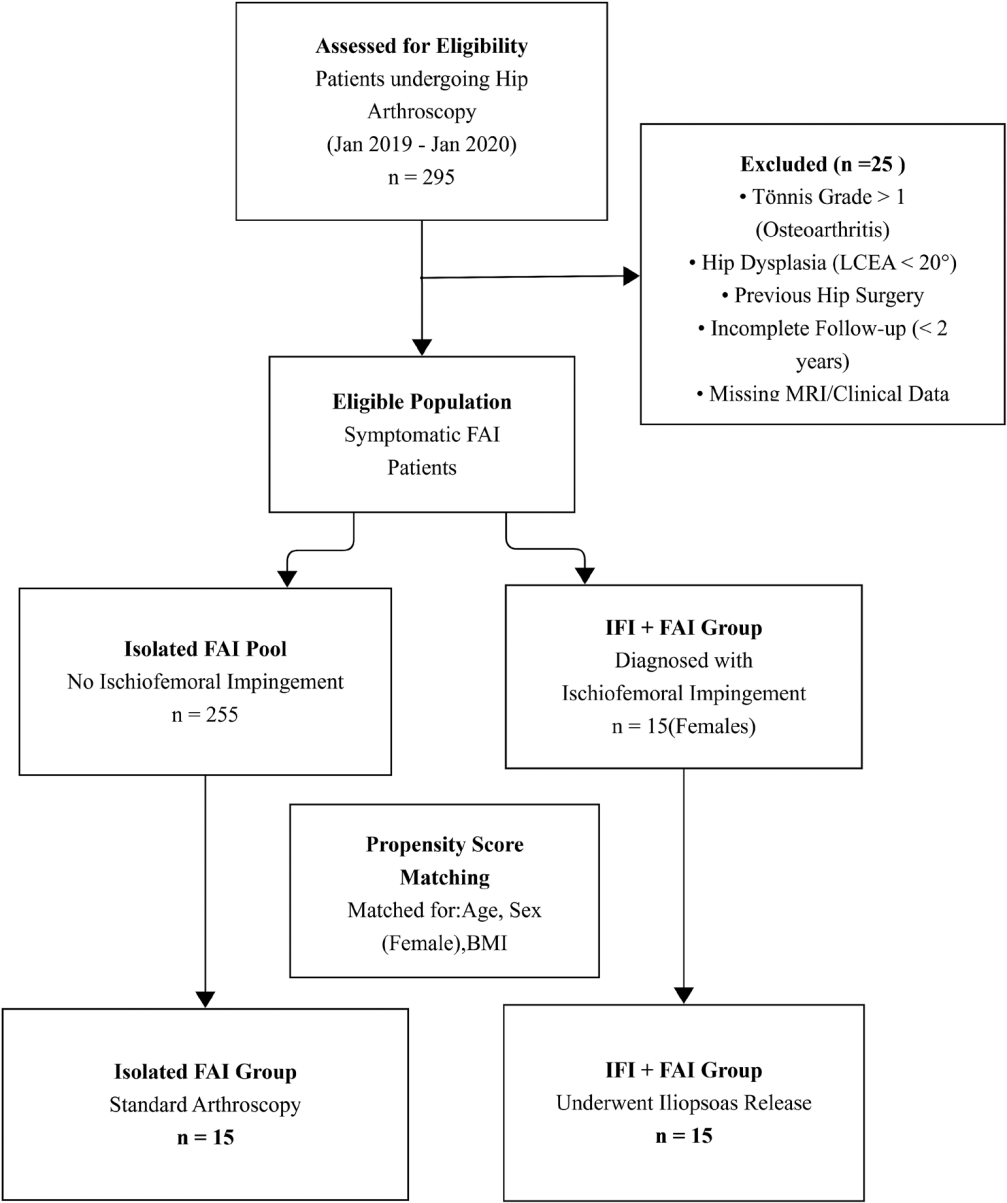
Patient selection and propensity score matching flowchart. FAI, femoroacetabular impingement; IFI, ischiofemoral impingement; PSM, propensity score matching.

Among the 30 patients ultimately included in the analysis: 15 were in the iliopsoas release combined with hip impingement group and 15 were in the isolated hip impingement group shown in Figure 4.

### 3.1 Demographic Data and Radiographic Findings

A total of 30 patients (30 hips) were included in the final analysis, consisting of 15 female patients in the IFI + FAI group and 15 propensity‐matched controls in the Isolated FAI group. All patients were female. There were no significant differences between the two groups regarding age at surgery (*p* = 0.75), body mass index (*p* = 0.73), or follow‐up duration (26.5 ± 4.2 months for the IFI + FAI group vs. 26.1 ± 3.8 months for the Isolated FAI group; *p* = 0.78) (Table 1).

**TABLE 1:** Demographic and radiographic data of patients included in study.

Variables	IFI + FAI group (*n* = 15)	Isolated FAI group (*n* = 15)	*p*
Age (range), years	45.4 ± 2.6 (30–55)	45.2 ± 3.1 (28–55)	0.85
Sex (female, *n* (%))	15 (100%)	15 (100%)	1
BMI (kg/m^2^)	21.8 ± 0.7	22.0 ± 0.8	0.45
Follow‐up (range), *m*	26.5 ± 4.2 (24.2–34.2)	26.1 ± 3.8 (24–35.6)	0.78
Side (left/right)	8/7	7/8	0.72
Alpha angle (°)	51.7 ± 1.4	52.1 ± 1.6	0.45
LCEA (°)	34.6 ± 2.4	34.2 ± 2.1	0.62
Neck‐Shaft angle (NSA, °)	134.9 ± 2.5	128.5 ± 3.2	< 0.001*
IFS (mm)	8.7 ± 1.5	22.4 ± 3.1	< 0.001*
QFS (mm)	5.1 ± 1.1	15.6 ± 2.2	< 0.001*

*Note:* Values are presented as mean ± standard deviation (range) or number (percentage).

Abbreviations: FAI, femoroacetabular impingement; IFI, ischiofemoral impingement; IFS, ischiofemoral space; LCEA, lateral center‐edge angle; NSA, neck‐shaft angle; QFS, quadratus femoris space.

* Indicates *p* < 0.05.

Radiographic evaluation demonstrated comparable cam and pincer morphology between groups, with no significant differences in the lateral center‐edge angle (LCEA; *p* = 0.82), Tönnis angle (*p* = 0.85), or alpha angle (*p* = 0.80). However, the IFI + FAI group exhibited specific anatomical features associated with ischiofemoral impingement. The neck‐shaft angle (NSA) was significantly greater in the IFI + FAI group compared with the Isolated FAI group (134.9° ± 2.5° vs. 128.5° ± 3.2°; *p* < 0.001). Furthermore, the IFI + FAI group demonstrated significantly narrowed ischiofemoral space (IFS; 8.7 ± 1.5 mm vs. 22.4 ± 3.1 mm; *p* < 0.001) and quadratus femoris space (QFS; 5.1 ± 1.1 mm vs. 15.6 ± 2.2 mm; *p* < 0.001).

### 3.2 Intraoperative Findings and Surgical Procedures

Intraoperative findings and surgical procedures are detailed in Table 2. There were no significant differences between the matched groups regarding the prevalence of labral tears, cartilage damage, or the distribution of cam and pincer deformities. Similarly, the rates of standard FAI procedures—including labral repair, femoroplasty, acetabuloplasty, and subspine decompression—were comparable between groups (*p* > 0.05). The only significant surgical difference was the treatment for ischiofemoral impingement; 100% of patients in the IFI + FAI group underwent endoscopic iliopsoas release and lesser trochanteroplasty, whereas no patients in the Isolated FAI group underwent these procedures (*p* < 0.001).

**TABLE 2:** Intraoperative findings and procedures.

Intraoperative findings/procedures	IFI + FAI group (*n* = 15)	Isolated FAI group (*n* = 15)	*p*
*Intraoperative findings*			
Labral tear (*n*, %)	15 (100%)	14 (93.3%)	1
Cartilage damage (ALAD grade > 2)	4 (26.7%)	3 (20.0%)	1
Cam deformity	15 (100%)	15 (100%)	1
Pincer deformity	12 (80%)	11 (73.3%)	1
*Surgical procedures*			
Iliopsoas release	15 (100%)	0 (0%)	< 0.001*
Lesser trochanter resection	15 (100%)	0 (0%)	< 0.001*
Acetabuloplasty	15 (100%)	15 (100%)	1
Femoroplasty	15 (100%)	15 (100%)	1
Labral repair	14 (93.3%)	15 (100%)	1
Synovectomy	14 (93.3%)	13 (86.7%)	1
Subspine decompression	8 (53.3%)	5 (33.3%)	0.462
Gluteus medius repair	2 (13.3%)	1 (6.7%)	1
Capsular closure	15 (100%)	15 (100%)	1

*Note:* Values are presented as Count (%). *p*‐values were calculated using the Fisher's exact test.

Abbreviation: ALAD, Acetabular Labrum Articular Disruption grade.

* Indicates statistically significant difference (*p* < 0.05).

### 3.3 MRI Evaluation of Muscle Volume

Quantitative MRI analysis revealed significant differences in muscle volume changes between the two groups (Table 3). Preoperatively, there were no significant differences in the muscle volume index of the iliopsoas or quadratus femoris between the IFI + FAI and Isolated FAI groups (*p* > 0.05).

**TABLE 3:** Comparison of normalized muscle volumes at 2‐year follow‐up.

	IFI + FAI group (*n* = 15)	Isolated FAI group (*n* = 15)	*p*
*Normalized iliopsoas volume*			
Preoperative	2129.9 ± 444.2	2110.5 ± 430.1	0.90
Postoperative	1587.4 ± 539.0	2085.1 ± 415.6	< 0.001*
Volume change (%)	−25.5 ± 8.5	−1.2 ± 3.5	< 0.001*
*Normalized quadratus femoris volume*			
Preoperative	344.0 ± 125.5	350.2 ± 118.4	0.89
Postoperative	384.1 ± 160.3	348.5 ± 120.1	0.49
Volume change (%)	+11.6 ± 4.1	−0.5 ± 2.1	< 0.001*

*Note:* Data are presented as mean ± standard deviation. Volumes are normalized to BMI (mm^3^/kg/m^2^).

* Indicates *p* < 0.05.

Postoperatively, the iliopsoas muscle in the IFI + FAI group demonstrated a significant volume reduction, decreasing from 2129.9 ± 444.2 mm^3^ to 1587.4 ± 539.0 mm^3^ (*p* = 0.008). This corresponded to a mean atrophy rate of 25.5%. In contrast, the iliopsoas volume in the Isolated FAI group showed no significant change (−1.2% ± 3.5%; *p* > 0.05). The difference in the magnitude of volume change between the two groups was statistically significant (*p* < 0.001).

Regarding the QF muscle, the IFI + FAI group exhibited a significant volume increase of 11.6% postoperatively (from 344.0 ± 125.5 mm^3^ to 384.1 ± 160.3 mm^3^), whereas the Isolated FAI group showed a negligible change of −0.5% (*p* < 0.001 for between‐group difference). Additionally, the IFI + FAI group demonstrated a significant increase in both IFS and QFS postoperatively compared with preoperative values (*p* < 0.001).

### 3.4 Patient‐Reported Outcomes

PROs and psychometric thresholds are summarized in Table 4 and Table 5. Preoperatively, there were no significant differences between groups in mHHS, HOS‐ADL, HOS‐SS, iHOT‐12, or VAS scores (*p* > 0.05), indicating comparable baseline status.

**TABLE 4:** Patient‐reported outcomes.

Outcome measure	IFI + FAI group (*n* = 15)	Isolated FAI group (*n* = 15)	*p*
*mHHS*			
Preoperative	56.1 ± 16.7	58.2 ± 14.5	0.72
Postoperative	84.2 ± 15.9	86.5 ± 12.1	0.65
*HOS‐ADL*			
Preoperative	59.8 ± 17.4	61.5 ± 15.2	0.77
Postoperative	77.1 ± 19.2	82.4 ± 14.8	0.38
*HOS‐SS*			
Preoperative	35.2 ± 12.5	38.4 ± 11.8	0.45
Postoperative	62.5 ± 18.4	78.2 ± 15.6	0.01*
*iHOT‐12*			
Preoperative	44.5 ± 23.4	46.8 ± 21.2	0.79
Postoperative	79.1 ± 19.9	90.5 ± 12.4	0.04*
*VAS pain*			
Preoperative	5.8 ± 1.7	5.6 ± 1.5	0.75
Postoperative	1.9 ± 2.2	1.5 ± 1.8	0.56

*Note:* Data are mean ± SD. *p* values compare IFI + FAI versus Isolated FAI groups.

Abbreviations: ADL, activities of daily living; FAI, femoroacetabular impingement; HOS, Hip Outcome Score; IFI, ischiofemoral impingement; iHOT‐12, International Hip Outcome Tool‐12; mHHS, modified Harris Hip Score; VAS, Visual Analog Scale.

* Indicates statistically significant difference (*p* < 0.05).

**TABLE 5:** MCID, PASS, and SCB attainment at minimum of 2 years after surgery.

	IFI + FAI group (*n* = 15)	Isolated FAI group (*n* = 15)	*p*
*MCID*			
HOS‐ADL	8 of 15 (53)	11 of 15 (73)	0.46
HOS‐SS	8 of 15 (53)	12 of 15 (80)	0.25
mHHS	9 of 15 (60)	11 of 15 (73)	0.7
iHOT‐12	9 of 15 (60)	11 of 15 (73)	0.7
*PASS*			
HOS‐ADL	7 of 15 (47)	10 of 15 (67)	0.46
HOS‐SS	7 of 15 (47)	11 of 15 (73)	0.27
mHHS	8 of 15 (53)	10 of 15 (67)	0.71
iHOT‐12	8 of 15 (53)	10 of 15 (67)	0.71
*SCB*			
HOS‐SS	5 of 15 (33)	12 of 15 (80)	0.02*
iHOT‐12	5 of 15 (33)	12 of 15 (80)	0.02*

*Note:* Data are *n* (%). Thresholds: mHHS (MCID 8, PASS 74); HOS‐ADL (MCID 9, PASS 87); HOS‐SS (MCID 6, PASS 75, SCB > 75); iHOT‐12 (MCID 13, PASS 63, SCB > 89).

Abbreviations: ADL, activities of daily living; FAI, femoroacetabular impingement; HOS, Hip Outcome Score; IFI, ischiofemoral impingement; iHOT‐12, International Hip Outcome Tool‐12; MCID, minimal clinically important difference; mHHS, modified Harris Hip Score; PASS, patient acceptable symptom state; SCB, substantial clinical benefit.

* Indicates statistically significant difference (*p* < 0.05).

At final follow‐up (mean 26.3 months; range, 24.0–35.6 months), both groups demonstrated significant improvements in all outcome measures compared with baseline values (*p* < 0.001). Outcomes were comparable regarding pain and daily function. No significant intergroup differences were found in postoperative mHHS (84.2 ± 15.9 vs. 86.5 ± 12.1; *p* = 0.65), HOS‐ADL (77.1 ± 19.2 vs. 82.4 ± 14.8; *p* = 0.38), or VAS scores (1.9 ± 2.2 vs. 1.5 ± 1.8; *p* = 0.56). Consistent with this, MCID and PASS attainment rates were similar between groups. For instance, PASS attainment for mHHS was 53% in the IFI + FAI group versus 67% in the Isolated FAI group (*p* = 0.71).

However, significant disparities emerged in high‐demand activities. The IFI + FAI group had significantly lower postoperative HOS‐SS (62.5 ± 18.4 vs. 78.2 ± 15.6; *p* = 0.01) and iHOT‐12 scores (79.1 ± 19.9 vs. 90.5 ± 12.4; *p* = 0.04). This was further highlighted by SCB analysis. A significantly lower proportion of patients in the IFI + FAI group achieved the SCB for HOS‐SS (33% vs. 80%; *p* = 0.02) and iHOT‐12 (33% vs. 80%; *p* = 0.02). There were no intraoperative or postoperative complications, such as heterotopic ossification, femoral head osteonecrosis, or excessive bleeding, and postoperative MRI confirmed the effective resection of the lesser trochanter in all cases.

### 3.5 Isokinetic Muscle Strength Evaluation

**FIGURE 5:**
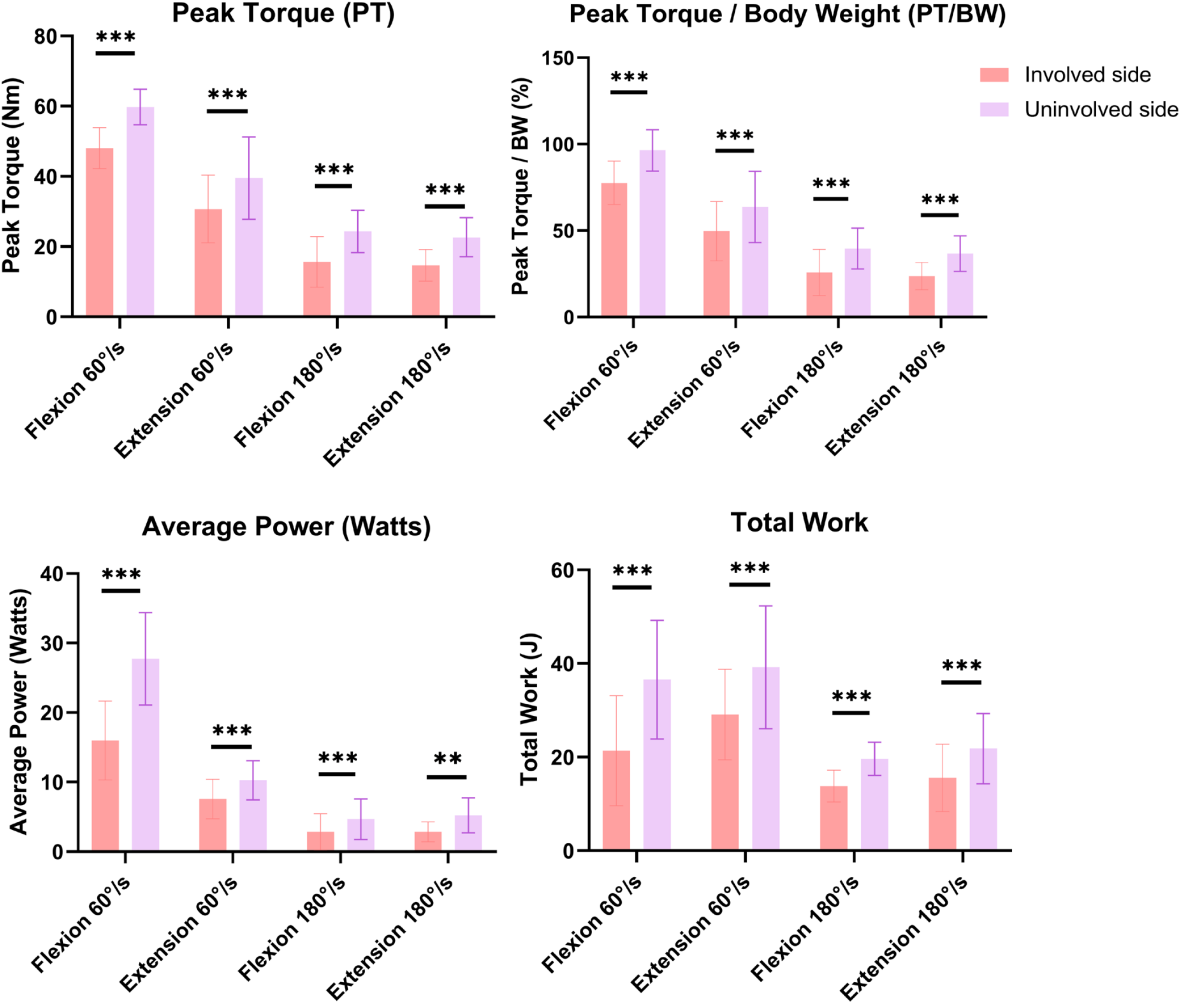
Comparison of isokinetic muscle performance between involved and uninvolved hips at 60°/s and 180°/s. Error bars represent standard deviation. ***p < 0.001.

For the 15 patients in the IFI + FAI group, isokinetic muscle strength testing was performed at the final follow‐up to compare the operative limb with the contralateral healthy limb (Figure 5; Table S1). The involved hip demonstrated significantly lower values across all measured parameters—Peak Torque, Normalized peak torque/body weight (PT/BW), Total Work, and Average Power—for both flexion and extension movements (*p* < 0.001 for all comparisons).

At low velocity (60°/s), the normalized peak torque (PT/BW) for hip flexion was 77.6% ± 12.6% on the involved side compared with 96.4% ± 11.9% on the uninvolved side (*p* < 0.001). Similarly, hip extension strength was significantly lower on the involved side compared with the uninvolved side (49.6% ± 17.1% vs. 63.7% ± 20.6%; *p* < 0.001).

At high velocity (180°/s), the average power output for flexion was 2.9 ± 2.6 W on the involved side versus 4.7 ± 2.9 W on the uninvolved side (*p* < 0.001). The extension power was also significantly lower on the involved side (2.9 ± 1.4 W) compared with the uninvolved side (5.2 ± 2.5 W) (*p* < 0.001).

### 3.6 MRI Assessment of Fatty Infiltration

Preoperatively, mean Goutallier grades were consistently low (< 0.4) at all three standardized levels, with no significant differences observed between the IFI + FAI and Isolated FAI groups (*p* > 0.05; Table 6).

**TABLE 6:** MRI evaluation of fatty infiltration (Goutallier grade).

MRI level		IFI + FAI group (*n* = 15) mean ± SD	Isolated FAI group (*n* = 15) mean ± SD	*p* ^a^
Level 1 (Medial Ilium)	Preoperative	0.20 ± 0.56	0.20 ± 0.41	0.88
	Postoperative	0.27 ± 0.59	0.27 ± 0.46	0.92
	*p* value^b^	0.34	0.34	
Level 2 (Femoral Head)	Preoperative	0.33 ± 0.49	0.27 ± 0.46	0.68
	Postoperative	0.40 ± 0.51	0.33 ± 0.49	0.72
	*p* value^b^	0.34	0.34	
Level 3 (Lesser Trochanter)	Preoperative	0.07 ± 0.26	0.13 ± 0.35	0.56
	Postoperative	0.13 ± 0.35	0.13 ± 0.35	0.99
	*p* value^b^	0.34	0.99	

*Note:* Data are presented as mean ± standard deviation. Level 1: Medial ilium; Level 2: Medial femoral head and neck; Level 3: Lesser trochanter.

^a^ Calculated using the Mann–Whitney U test (Comparison between groups).

^b^ Calculated using the Wilcoxon signed‐rank test (Comparison between preoperative and postoperative values).

Postoperatively, no significant intergroup differences were found in fatty infiltration scores. At Level 3 Lesser Trochanter, the mean Goutallier grade was 0.13 ± 0.35 in the IFI + FAI group compared with 0.13 ± 0.35 in the Isolated FAI group (*p* = 0.99). Similarly, no significant differences were detected at Level 1 (*p* = 0.92) or Level 2 (*p* = 0.72).

Within the IFI + FAI group, postoperative scores at Level 1 (*p* = 0.34), Level 2 (*p* = 0.34), and Level 3 (*p* = 0.34) showed no statistically significant change compared with preoperative values. Regarding the reliability of the assessment, the weighted Kappa values for the Goutallier classification ranged from 0.89 to 0.90 across all evaluated levels.

## 4 Discussion

### 4.1 Clinical Outcomes and Daily Function

The principal finding of this study is that arthroscopic management of concomitant IFI and FAI, which necessitates iliopsoas release, provides significant pain relief and functional restoration. Consistent with previous studies [9, 29, 33], our patients achieved significant improvements in PROs [34]. However, our objective testing highlights a critical physiological trade‐off: while the procedure effectively restores activities of daily living, it results in a significant reduction in muscle volume and maximal muscle strength, which may limit high‐demand athletic performance [35].

### 4.2 Muscle Morphology: Volumetry and Fatty Infiltration

Our 3D volumetric analysis confirmed a 25.5% reduction in iliopsoas muscle volume postoperatively, a finding that aligns with Brandenburg et al. [14], who reported similar atrophy rates. This volume loss is likely a mechanical consequence of the tenotomy: the retracted muscle belly shortens, resulting in reduced overall volume.

Importantly, despite this volume loss, our MRI results using the Goutallier classification [16] showed no significant fatty infiltration (Grade < 1). This distinction is critical: the muscle became “smaller” but not “fatter” or degenerated. This suggests that the atrophy observed is disuse‐ or length‐dependent, rather than the irreversible fatty degeneration described in rotator cuff tears [36, 37].

### 4.3 Strength Deficits and Functional Implications

A key finding of this study is the significant strength deficit observed in isokinetic testing. Unlike the preservation of muscle quality, the involved hips demonstrated significantly lower peak flexion torque compared to the uninvolved contralateral side (*p* < 0.001). This finding corroborates the concerns raised by Brandenburg et al. [14] who noted hip flexion weakness following release.

Paradoxically, despite this objective weakness, our patients reported excellent HOS‐ADL scores. This discrepancy can be explained by the “functional redundancy” of the hip flexor complex. Daily activities such as walking or climbing stairs typically utilize only a fraction of maximal muscle torque. As noted by Ilizaliturri et al. [30] and others, the remaining iliopsoas unit, along with synergistic compensation from the rectus femoris and tensor fasciae latae, provides sufficient power for these routine tasks. The significant improvement in ADL scores likely reflects the resolution of pain, which allows patients to perform daily activities comfortably despite the lower absolute strength ceiling.

### 4.4 Clinical Implications and Surgical Decision‐Making

In contrast to daily activities, the significant strength deficit becomes relevant during high‐demand sports that require maximal explosive power. This explains why, in our cohort and similar studies, the improvement in sport‐specific scores (e.g., HOS‐SS) often lags ADL scores. The loss of 26% of muscle volume and the associated drop in peak torque represents a reduction in the “physiological reserve” required for sprinting or high‐performance athletics. Therefore, while iliopsoas lengthening performed for appropriate indications generally does not appear to compromise mid‐term patient‐reported outcomes or increase revision/total hip arthroplasty (THA) conversion rates [13, 38, 39], surgeons should explicitly counsel high‐level athletes about the potential trade‐off in top‐end explosive performance.

The decision to perform a release must therefore balance these factors. In our cohort, the release was performed for mechanical decompression of the ischiofemoral space and to resolve painful snapping. As noted by El Bitar et al. [11], arthroscopic fractional lengthening is effective for resolving internal snapping and improving clinical scores when pathologic conflict is present. The resolution of the “nutcracker” impingement [2, 4] allows patients to return to a pain‐free daily life, even if their maximal athletic envelope is subtly compromised [40].

### 4.5 Strengths and Limitations

Methodological strengths of this study include the use of propensity score matching to minimize selection bias, advanced 3D volumetric muscle reconstruction, and the inclusion of objective isokinetic strength testing alongside clinical outcomes. Nevertheless, this study is not without limitations. First, its retrospective design introduces selection bias, although propensity matching was employed. Second, while we utilized advanced 3D reconstruction techniques similar to those described by Kikinis [25] and Leijendekkers [41, 42], the sample size remains relatively small. Third, all patients in our cohort were female. While this limits the generalizability of our findings to the male population, it reflects the known epidemiological characteristics of IFI, which overwhelmingly affects women due to pelvic anatomy. Fourth, the Iliacus and Psoas Major were segmented as a combined unit. Due to their distal fusion at the lesser trochanter and MRI resolution limits, distinct segmentation was not feasible, preventing the assessment of differential muscle atrophy. Fifth, concomitant procedures represent a confounding factor. While the matched control group allowed us to isolate the tenotomy's effect on muscle volume, distinguishing the independent clinical benefit of the release from the overall FAI treatment remains challenging.

## 5 Conclusion

In this retrospective study of female patients, arthroscopic iliopsoas release for concomitant IFI and FAI yielded significant clinical improvement and pain resolution without inducing fatty degeneration. However, acknowledging the limitations of sample size and concomitant procedures, the objective strength deficits observed in this cohort serve as a critical caution. Consequently, while the procedure is effective for symptom management, surgeons must carefully counsel high‐demand athletes regarding the potential compromise in explosive power.

## Author Contributions

Y.‐P.D. and H.S. contributed equally to this work as co‐first authors. Y.‐P.D., H.S., H.‐J.H., Y.X., X.‐D.J., and J.‐Q.W. were involved in the study design, data collection, and analysis. Y.‐P.D. and H.S. drafted the manuscript. Y.X., X.‐D.J., and J.‐Q.W. provided critical revision of the manuscript for important intellectual content and gave final approval of the version to be published.

## Funding

This work was supported by Natural Science Foundation of Beijing Municipality (7242167).

## Conflicts of Interest

The authors declare no conflicts of interest.

## Supporting information


**Supplementary Material S1:** 3D reconstruction of iliopsoas muscle (green) and Quadratus femoris muscle (blue).

**Table S1:** Comparison of isokinetic muscle strength metrics between involved and uninvolved hips.

## Data Availability

The data that support the findings of this study are available on request from the corresponding author. The data are not publicly available due to privacy or ethical restrictions.
