# Supplementary material for: Preserved Daily Function Despite Significant Muscle Atrophy and Strength Deficits: A Matched‐Cohort Study of Iliopsoas Release for Ischiofemoral Impingement
Source: Orthop Surg. 2026 Apr 22;18(6):1241–53. doi: 10.1111/os.70295 (PMC13238807; doi:10.1111/os.70295)
Supplement: Supplementary file 1 — Supplementary Material S1: 3D reconstruction of iliopsoas muscle (green) and Quadratus femoris muscle (blue). [file OS-18-1241-s002.docx]

**Supplementary Material 1:3D reconstruction of iliopsoas muscle(green) and Quadratus femoris muscle(blue)**
