# Supplementary material for: Preserved Daily Function Despite Significant Muscle Atrophy and Strength Deficits: A Matched‐Cohort Study of Iliopsoas Release for Ischiofemoral Impingement
Source: Orthop Surg. 2026 Apr 22;18(6):1241–53. doi: 10.1111/os.70295 (PMC13238807; doi:10.1111/os.70295)
Supplement: Supplementary file 2 — Table S1: Comparison of isokinetic muscle strength metrics between involved and uninvolved hips. [file OS-18-1241-s001.docx]

**Supplementary Table 1:** **Comparison of Isokinetic Muscle Strength Metrics Between Involved and Uninvolved Hips**

| Metric / Condition | Involved Side(n=15) | Uninvolved Side(n=15) | P Value |
| --- | --- | --- | --- |
| **Peak Torque (Nm)** |  |  |  |
| Flexion 60°/s | 48.0±5.8 | 59.8±5.1 | <.001 |
| Extension 60°/s | 30.7±9.7 | 39.5±11.7 | <.001 |
| Flexion 180°/s | 15.7±7.2 | 24.3±6.0 | <.001 |
| Extension 180°/s | 14.6±4.5 | 22.7±5.5 | <.001 |
| **Peak Torque / BW (%)** |  |  |  |
| Flexion 60°/s | 77.6±12.6 | 96.4±11.9 | <.001 |
| Extension 60°/s | 49.6±17.1 | 63.7±20.6 | <.001 |
| Flexion 180°/s | 25.8±13.3 | 39.6±11.8 | <.001 |
| Extension 180°/s | 23.7±7.9 | 36.7±10.2 | <.001 |
| **Total Work (J)** |  |  |  |
| Flexion 60°/s | 21.4±11.8 | 36.6±12.7 | <.001 |
| Extension 60°/s | 29.1±9.7 | 39.2±13.1 | <.001 |
| Flexion 180°/s | 13.8±3.4 | 19.6±3.5 | <.001 |
| Extension 180°/s | 15.6±7.2 | 21.8±7.5 | <.001 |
| **Average Power (Watts)** |  |  |  |
| Flexion 60°/s | 16.0±5.7 | 27.7±6.6 | <.001 |
| Extension 60°/s | 7.6±2.9 | 10.3±2.8 | <.001 |
| Flexion 180°/s | 2.9±2.6 | 4.7±2.9 | <.001 |
| Extension 180°/s | 2.9±1.4 | 5.2±2.5 | <.001 |

Note: Values are presented as mean ± standard deviation. Abbreviations: BW, body weight; Nm, Newton-meters; J, Joules. P-values were calculated using the paired samples t-test. Comparisons were made between the involved and uninvolved hips within the IFI group (n=15). The uninvolved contralateral side served as the control, as isokinetic muscle strength data were not collected for the Isolated FAI group.
